# Dark Sweet Cherry (*Prunus avium*) Anthocyanins Suppressed ERK1/2-Akt/mTOR Cell Signaling and Oxidative Stress: Implications for TNBC Growth and Invasion

**DOI:** 10.3390/molecules27217245

**Published:** 2022-10-25

**Authors:** Ana Carolina Silveira Rabelo, Susanne U. Mertens-Talcott, Boon P. Chew, Giuliana Noratto

**Affiliations:** 1 Department of Food and Experimental Nutrition, Faculty of Pharmaceutical Sciences, University of São Paulo, São Paulo 05508 270, Brazil; 2 Department of Food Science and Technology, Texas A&M University, College Station, TX 77843-2253, USA

**Keywords:** 4T1-CRL-2539, anthocyanins, dark sweet cherries, polyphenols, metastasis, angiogenesis

## Abstract

This study aimed to assess dark sweet cherry (DSC) total polyphenols (WE) and anthocyanins (ACN) against metastatic breast cancer (BC). The WE and ACN anticancer activity and underlying mechanisms were assessed in vitro using 4T1 BC cells. A pilot study using a BALB/C mouse syngeneic model bearing 4T1 tumors assessed the anti-metastatic potential of ACN in vivo. ACN inhibited cell viability with higher potency than WE and reduced reactive oxygen species (ROS) (IC_50_ = 58.6 µg cyanidin 3-glucoside equivalent (C3G)/mL or 122 µM). ACN induced p38 stress-related intrinsic apoptosis, leading to caspase-3 cleavage and total PARP decrease. ACN suppressed ERK1/2 and Akt/mTOR signaling pathways, which are abnormally activated in BC and promote motility and invasion. This was consistent with suppression of VCAM-1 mRNA, Scr phosphorylation and 88.6% reduction of cells migrating to wounded area. The pilot in vivo results supported the ACN-mediated suppression of angiogenesis in tumors and lungs. ACN also lowered Cenpf mRNA in lungs, associated with lung metastasis lesions and poor survival. Results demonstrated the dual Akt-ERK inhibitory role of ACN and suppression of their downstream pro-invasive targets. These results encourage a larger scale in vivo study to confirm that ACN may help to fight BC invasion and metastasis.

## 1. Introduction

BC has become the world’s most commonly diagnosed cancer, with more than 2.3 million women diagnosed and 685,000 breast cancer deaths recorded in 2020 [1]. In the U.S., about 13% women (1 in 8) will develop invasive BC over the course of their lifetimes, with approximately 287,850 new cases of invasive BC expected to be diagnosed in 2022 [2]. Worldwide, between 20% and 30% of women with BC will develop distant metastasis, which may lead to approximately 400,000 to 500,000 deaths per year [3]. For this reason, a high priority in the fight against BC is to investigate the efficacy of chemopreventive dietary strategies and improve the understanding of underlying target mechanisms.

The murine 4T1 BC cell line has been extensively used in in vitro and in vivo research models to investigate the tumor cell signaling pathways involved in oncogenesis, tumor progression, and drug resistance. The most studied pathway is that of mitogen-activated protein kinase (MAPK) because it is an important link between extracellular signals and their intracellular responses. MAPK is known to be a complex interconnected signaling cascade with frequent involvement in oncogenesis, tumor progression, and drug resistance. The three main MAPKs are extracellular signal-regulated kinases (ERK), c-Jun N-terminal kinases (JNKs), and p38. The ERK pathway plays a key role in triple negative breast cancer (TNBC) progression [4]. The ERK pathway is activated by growth factors occurring through the binding of receptor tyrosine kinases (RTKs) at the cell membrane level. The cell signal activation by phosphorylation of cytosolic intermediates represented by the RAS superfamily leads to activation of the downstream effector RAF, which is responsible for the pathway progression by activating MAP kinase-ERK kinase (MEK) and ERK1/2. Once this cell signal is activated, ERK1/2 phosphorylates numerous target proteins in the cytoplasm and then migrates to the nucleus to phosphorylate several transcription factors for DNA synthesis and cell cycle progression [5].

The p38 MAPK pathway instead responds mainly to environmental stress signals and inflammatory stimuli, maintaining cellular homeostasis [6]. The dual role of p38 implies that it regulates cell death or cell survival, being highly context dependent.

Targeting the ERK pathway in cancer cells is not simple because cells develop resistance by activating compensatory feedback loops such as the phosphatidylinositol-3-kinase (PI3K)—protein kinase B (Akt)—mammalian target of rapamycin (mTOR) (Akt/mTOR) signaling. The PI3K/Akt/mTOR pathway is one of the most common genomic abnormalities in TNBC [7]. Akt overexpression leads to protein synthesis and cell growth by activating the downstream effector mTOR through tuberous sclerosis complex 1 and 2 (TSC1/2) and ribosomal protein S6 (RPS6) [8].

Another altered mechanism in TNBC is the epithelial–mesenchymal transition (EMT), where polarized epithelial cells are converted into non-polarized mesenchymal cells, leading to invasion and motility [9]. During this process, there is an overexpression of Src, a kinase protein associated with integrin signaling and Akt activation. Consequently, the Akt pathway also contributes to EMT to promote motility and invasion, protein synthesis and phenotype transformation [10].

An Akt/mTOR–ERK crosstalk occurs in TNBC, leading to aberrant signaling activation [5,11]. Therefore, the combination of ERK and Akt/mTOR pathway inhibition may contribute to an effective targeted therapy to fight TNBC [12].

Even though every model has its limitations due to the genetic and phenotypic heterogeneity of human breast cancers, 4T1 cells can be used in BALB/C mouse as a syngeneic model due to its ability to metastasize spontaneously from the orthotopic site to the lung, liver, bone, and brain via the hematogenous route [13], mimicking stage IV human BC [14]. This in vivo model has shown to be of great advantage because both the tumor microenvironment and the transplanted tumors are from the same species and have similar genetic backgrounds, which favor the close interaction between tumor cell and the host stroma [15].

Epidemiological studies have suggested an inverse correlation between the intake of fruits and vegetables and the incidence of BC [16]. The potential underlying mechanisms relate to the phytochemicals (e.g., polyphenols in berries) and their ability to counteract oxidative stress and inflammation, as well as the downregulation of proteins that promote BC proliferation and invasion [17]. The BC chemopreventive activity of DSC polyphenol fraction enriched in anthocyanins (ACN) proved to be effective in inhibiting the growth of the most aggressive MDA-MB-453 and MDA-MB-231 cells and the less aggressive BT-474 BC cells, while the mixture of total polyphenols (WE) inhibited MDA-MB-453 [18]. Both WE and ACN did not promote toxicity to the non-cancerous MCF-10A breast epithelial cells [18].

The underlying molecular mechanisms modulated by ACN in MDA-MB-453 cells involved the induction of the intrinsic apoptotic pathway as a primary pro-apoptotic mechanism and the extrinsic apoptotic pathway mediated by caspase-8 cleavage as a secondary pro-apoptotic mechanism. In addition, the ACN-mediated inhibition of Akt signaling and phosphoinositide-specific phospholipase C (PLCγ-1) activation contributed to impair cell wound healing [19]. The in vivo antitumor potential of ACN was later confirmed in MDA-MB-453 xenografted tumor protein expression analyses showing 66 proteins associated with poor BC prognosis expressed or differentially up-regulated (*p* < 0.05) only in the untreated control group compared to the ACN-treated group [20]. However, the cell line and animal model in this study did not allow assessment of the anti-invasive and anti-metastatic potential of ACN.

The aim of this study was to assess WE and ACN extracts against 4T1 cells, which mimic stage IV human BC, with the focus on underlying molecular targets that contribute to mobility, invasion, and metastasis. A complementary objective was to perform an in vivo pilot study to collect preliminary data and to identify caveats and pitfalls in order to design a larger scale in vivo study.

## 2. Results

### 2.1. WE and ACN Fractions Reduced 4T1 BC Cell Viability and Decreased ROS 

**Figure 1:**
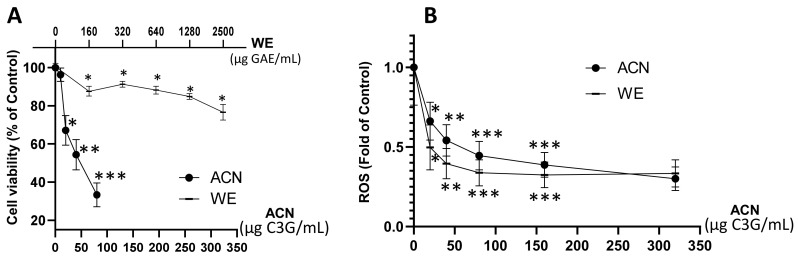
DSC extract enriched in ACN reduced 4T1 BC cell viability and ROS production. 4T1 cell viability (**A**). Cells were treated with ACN (10–80 μg C3G/mL) or WE (160–2500 μg GAE/mL, ~20–320 μg C3G/mL) for 48 h. Cell viability was assessed using the in vitro toxicology resazurin based assay kit. ROS levels (**B**). Cells were treated with ACN or WE (20–320 μg C3G/mL) for 48 h. Production of ROS was assessed with the Carboxy-H2DFFDA probe. RFU were normalized to cell density assessed using the Janus green reagent as detailed in the materials and methods section. Data are mean ± SEM (*n* ≥ 6). Significant difference was determined by ANOVA followed by Bonferroni test against untreated control (* *p* < 0.05, ** *p* < 0.01, *** *p* < 0.001).

Results showed that WE exerted a dose-dependent cell viability inhibition down to 76% of untreated control within a dose range of 160–2500 µg GAE/mL (~20–320 µg C3G/mL), while the ACN fraction suppressed cell viability down to 33% of untreated control within a dose range of 20–80 µg C3G/mL (Figure 1A). The dose of ACN needed to inhibit cell viability by 50% (IC_50_) was 58.6 µg C3G/mL (122 µM). These results proved the higher potency of ACN against 4T1 BC cells compared to WE, which contains a mixture of anthocyanins and other phenolic compounds present in DSC. The DSC concentrated juice used for this study was analyzed by HPLC-MS/MS [18]. It was found that the main compounds are hydroxycinnamic acids (698.96 ± 7.07 mg chlorogenic acid equivalent/L DSC concentrated juice), hydroxybenzoic acids (64.06 ± 9.80 mg GAE/L DSC concentrated juice), flavonols and other flavonoids (520.88 ± 7.99 mg quercetin-3-O-rutinoside equivalent/L DSC concentrated juice), and flavan-3-ols (1831.43 ± 93.79 mg epicatechin equivalent/L DSC concentrated juice) [18]. 

Anthocyanins in ACN and WE (dose range 20–320 µg C3G/mL) downregulated ROS production to 0.3 ± 0.07-fold and 0.33 ± 0.08-fold of untreated controls, respectively (Figure 1B). These results demonstrated that the anthocyanins present in both WE and ACN extracts decreased ROS, contributing to cell growth suppression.

Based on these results, the DSC fraction enriched in anthocyanins (ACN) at its IC_50_ dose (58.6 µg C3G/mL or 122 µM C3G) was chosen to investigate the underlying molecular mechanisms exerted in 4T1 cells.

### 2.2. ACN Induced Apoptosis in 4T1 BC Cells

**Figure 2:**
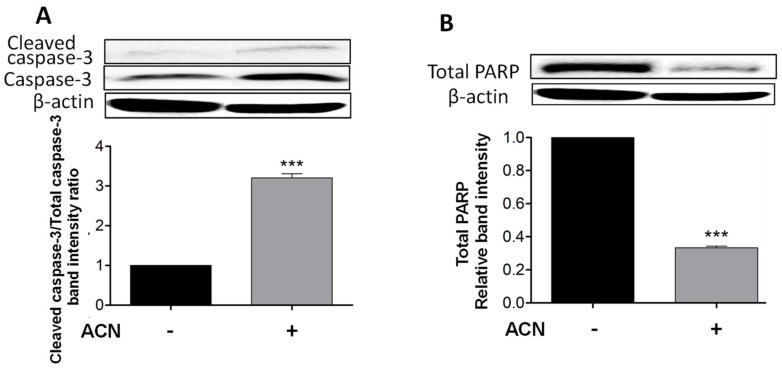
ACN induced apoptosis in 4T1 BC cells through a caspase-dependent pathway. Cleaved and total caspase-3 protein levels and ratio of cleaved caspase-3 to total caspase-3 (**A**). Total PARP protein levels (**B**). Cells starved overnight were treated with ACN (IC_50_) in 2.5% FBS supplemented culture medium for 48 h. Cell lysates were subjected to immunoblot analysis with primary and secondary antibodies as detailed in materials and methods. Western blots are representative of at least two independent experiments (upper panels). Band intensities of target proteins determined with ImageJ software were divided by band intensity of β-actin, and values were normalized to untreated control (lower panels). Data from relative band intensity are mean ± SEM. The unpaired *t* test with Welch’s correction was used to determine significance (*** *p* < 0.001).

Cleaved caspase-3 is a reliable marker for cell death along with total PARP-1 protein decrease, which were detected after 48 h of ACN treatment as shown in Figure 2A,B. Even though the timeline for apoptosis from initiation to completion can occur within 12–24 h, ACN treatment did not induce caspase-3 cleavage at 24 h (Supplementary Figure S1A) or decrease total PARP-1 levels (Supplementary Figure S1B).

Caspase-3 cleavage occurs downstream of both the extrinsic and intrinsic pathways and as executioner caspase. However, cleaved caspase-8 was not detected by Western blots, suggesting the mitochondrial pathway as the primary pro-apoptotic mechanism induced by ACN in 4T1 cells.

### 2.3. ACN Modulated ERK and p38 Signaling Pathways in 4T1 BC Cells

**Figure 3:**
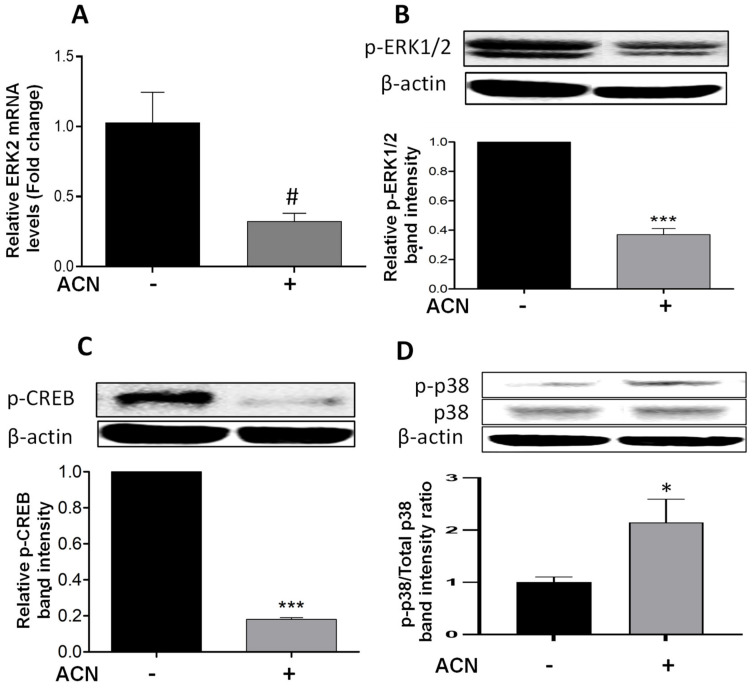
ACN downregulated ERK and its target CREB and induced the phosphorylation of stress-related p38. mRNA levels of ERK2 (**A**). Protein levels of p-ERK1/2 (**B**), p-CREB (**C**), and p-p38 and total p38 (**D**). The ERK2 mRNA levels were determined after 24 h treatment with ACN (IC_50_). Protein levels of p-ERK1/2 and p-CREB were determined after 48 h treatment with ACN. Protein levels of p-p38 and total p38 were determined after 24 h ACN treatment. Cells starved overnight were treated with ACN (IC_50_) in 2.5% FBS supplemented culture. Cell lysates were subjected to immunoblot analysis with primary and secondary antibodies as detailed in materials and methods. Western blots are representative of at least two independent experiments (upper panels). Band intensities of target proteins determined with ImageJ software were divided by band intensity of β-actin, and values were normalized to untreated control (lower panels). Data from relative band intensity are mean ± SEM. The unpaired *t* test with Welch’s correction was used to determine significance (*** *p* < 0.001; * *p* < 0.05, ^#^ *p* = 0.075).

Results showed that ACN downregulated ERK2 at the gene expression level to 0.32-fold of untreated control (Figure 3A) but did not reach significance (*p* = 0.075). The levels of ERK1/2 phosphorylated protein (p-ERK1/2) were reduced (Figure 3B). One of the mechanisms promoted by ERK1/2 is the phosphorylation of CREB in serine 133, activating this transcription factor [21]. Consistent with p-ERK1/2 downregulation, phosphorylated CREB (p-CREB) was also suppressed (Figure 3C).

The simultaneous induction of p38 and suppression of ERK activity has been reported as a mechanism to induce apoptosis in different cancer cell lines [22]. This was consistent with results showing the upregulation of p-p38 (Figure 3D), and the significant increase in p-p38/total p38 protein ratio (Figure 3D, lower panel). These results strongly suggest that p38 may be involved in stress-induced apoptosis activation [12]. In addition, the ACN-mediated suppression of p-CREB may have also contributed to induce the intrinsic apoptotic pathway [23].

### 2.4. ACN Downregulated Akt/mTOR Signaling Pathway in 4T1 BC Cells

**Figure 4:**
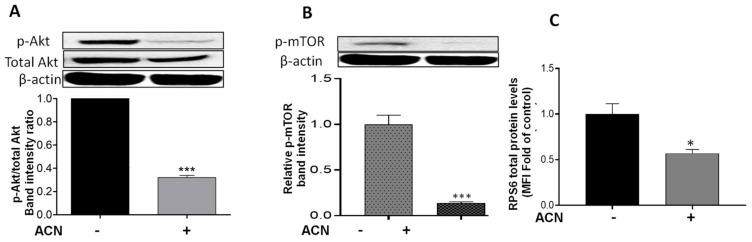
ACN downregulated Akt/mTOR pathway in 4T1 BC cells. Protein levels of p-Akt (**A**), p-mTOR (**B**), and RPS6 (**C**). Cells starved overnight were treated with ACN (IC_50_) in 2.5% FBS supplemented culture for 48 h. Protein levels were determined in cell lysates. Akt, p-Akt, and p-mTOR levels were determined by immunoblot analysis with primary and secondary antibodies as detailed in materials and methods. Western blots are representative of at least two independent experiments (upper panels). Band intensities of target proteins determined with ImageJ software were divided by band intensity of β-actin, and values were normalized to untreated control (lower panels). Protein levels of RPS6 were determined with Millipex MAP magnetic bead assay. The RPS6 mean fluorescent intensity (MFI) values of cell lysates were normalized to untreated controls. Data from MFI are mean (*n* = 3) ± SEM. The unpaired *t* test with Welch’s correction was used to determine significance (*** *p* < 0.001; * *p* < 0.05).

Results showed that Akt mRNA levels were not changed by ACN treatment (data not shown). However, ACN downregulated Akt protein activation by phosphorylation (Figure 4A). The Akt cell signaling downregulation was consistent with the lower p-Akt/total Akt protein ratio (Figure 4A, lower panel). Consistently, ACN downregulated mTOR phosphorylation (p-mTOR) (Figure 4B). Levels of full-length proteins within the PI3K/Akt/mTOR cell signaling pathway (illustrated in Supplementary Figure S2), assessed by multiplex immunoassays, remained without significant changes after ACN treatment for 24–48 h (Supplementary Table S1). Therefore, p-mTOR/total mTOR ratio decreased to 0.14-fold of untreated control (Supplementary Figure S3). RPS6 protein, also a downstream target of Akt (Figure S2), was downregulated by ACN treatment to 0.57 ± 0.08-fold of untreated control (Figure 4C).

### 2.5. ACN Reduced Cell Wound Healing and Invasive Potential

**Figure 5:**
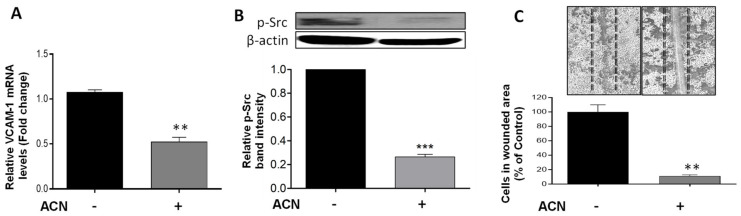
ACN downregulated VCAM-1 and p-Src involved in BC invasion and suppressed wound healing in 4T1 cells. VCAM-1 mRNA levels (**A**). The VCAM-1 mRNA levels were determined after 24 h treatment with or without ACN (IC_50_) as detailed in materials and methods. Protein levels of p-Src (**B**). Protein levels of target proteins were determined after 48 h ACN treatment in cell lysates by immunoblot analysis with primary and secondary antibodies as detailed in materials and methods. Western blots are representative of at least two independent experiments (upper panel). Band intensities of p-Src determined with ImageJ software were divided by band intensity of β-actin, and values were normalized to untreated control (lower panel). ACN-mediated suppression of wound healing (**C**). Quantification of wound healing was performed by counting the number of cells in scratched area after 48 h treatment with ACN (IC_50_) using ImageJ software. Data from mRNA are mean ± SEM (*n* = 3). Data from relative band intensity determined with ImageJ software are mean ± SEM. Data from wound healing assay are mean (*n* ≥ 4) ± SEM. The unpaired *t* test with Welch’s correction was used to determine significance (*** *p* < 0.001, ** *p* < 0.01).

Vascular cell adhesion molecule (VCAM)-1 has been implicated in cancer cell migration in part due to its link to the Src signaling activation. Results demonstrated that ACN downregulated VCAM-1 at the gene expression level to 0.52-fold of untreated control (*p* = 0.0019) (Figure 5A). This was accompanied by suppression of p-Src (Figure 5B). An important role of Src is to increase the strength of cell–cell adhesion to promote collective cell migration closely related to the epithelial–mesenchymal transition. Accordingly, the anti-invasive potential of ACN was further confirmed by the cell wound healing assay. Results showed a reduction in the number of cells that invaded the wounded area to 11.4 ± 2.9% that of control (Figure 5C).

### 2.6. Results from In Vivo Pilot Study Supported Mechanisms Targeted by ACN In Vitro 

Implanted tumors reached a volume of 1.6 ± 0.6 cm^3^ after 9 days of orthotopic injection before ACN treatment (150 mg C3G/kg/day) by gavage for 1 week. As expected, there was no difference between untreated controls and ACN regarding tumor volume at the end of the study (2.5 ± 0.008 cm^3^, and 2.2 ± 0.6 cm^3^, respectively). However, the weights of tumors in the ACN group tended to be lower than in untreated control group (1.85 ± 0.6 g, vs. 2.6 ± 0.8 g, respectively; *p* > 0.05), which may be related to less angiogenesis. 

### 2.7. ACN Reduced Angiogenesis in BC Tumors In Vivo

**Figure 6:**
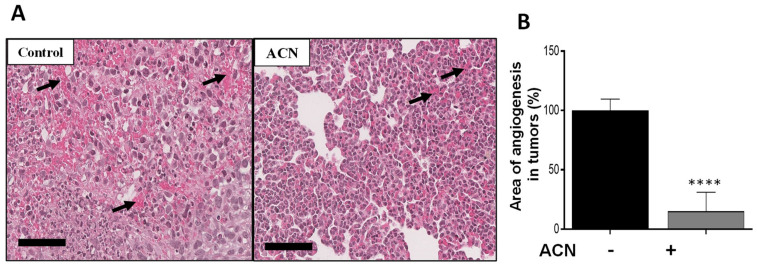
ACN decreased angiogenesis in tumor tissues. Photomicrographs of tumor tissues with arrows showing blood vessels (**A**). Quantification of angiogenesis areas in tumor tissues (**B**). Photomicrographs of tumor tissues (H&E, 28X) are representative of *n* ≥ 10 random areas per stained section (bar represents 70 μm). Quantification of angiogenesis area was determined with ImageJ software. Data are mean (*n* ≥ 50) ± SEM. The unpaired *t* test with Welch’s correction was used to determine significance (**** *p* < 0.0001).

Angiogenesis is characterized by the formation of new blood vessels, allowing the necessary supply of oxygen and nutrients for tumor growth. Histological analysis showed that ACN reduced the angiogenesis area in tumor tissues by 85 ± 1.4% (difference between means ± SEM) (Figure 6A,B). Preliminary results from levels of proteins in tumor tissues showed that expression of p-ERK1/2 was significantly downregulated, as well as that of p-CREB in the ACN group (Supplementary Figure S4). These preliminary results are consistent with the in vitro findings regarding ACN-mediated suppression of the p-ERK/p-CREB axis.

### 2.8. ACN Decreased Biomarkers of BC Metastasis in Lungs

**Figure 7:**
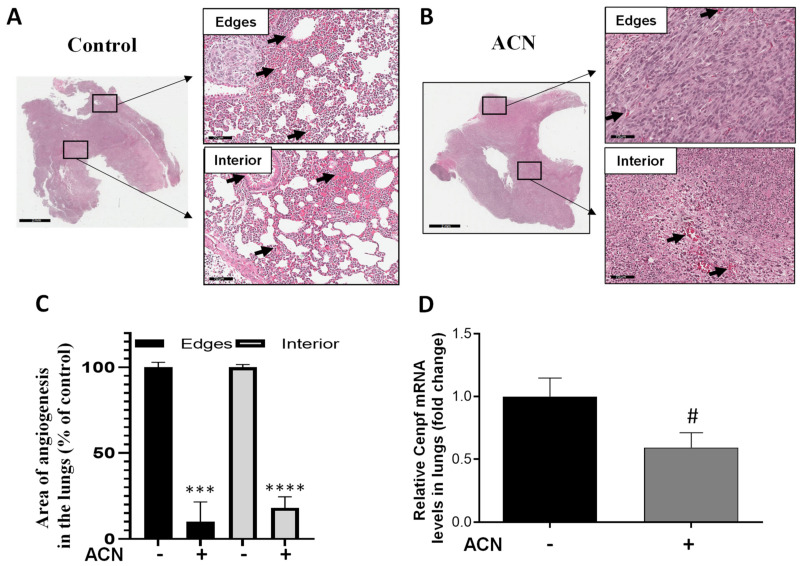
ACN decreased angiogenesis and Cenpf mRNA in lungs. Photomicrographs of lung tissues with arrows showing blood vessels in lungs of untreated control (**A**) and ACN-treated mice (**B**). Quantification of angiogenesis areas in edges and interior of lung tissues (**C**). Lung tissues were excised after 1 week ACN treatment (150 mg C3G/kg BW/day) as detailed in materials and methods. Photomicrographs of lung tissues (H&E, 30X) are representative of *n* ≥ 10 random areas per stained section (bar represents 70 μm). Quantification of angiogenesis area was determined with ImageJ software. Data are mean (*n* = 25 for untreated control and *n* = 12–14 for ACN) ± SEM. Cenpf mRNA levels in lung tissues (**D**). Lung tissues were ground with liquid nitrogen followed by mRNA extraction as detailed in materials and methods. Data from mRNA analysis are mean (*n* = 4) ± SEM. The unpaired *t* test with Welch’s correction was used to determine significance (**** *p* < 0.0001, *** *p* < 0.001, ^#^ *p* = 0.0768).

Areas showing angiogenesis in lung tissues were measured in edges and interior of H&E-stained sections. As shown in Figure 7A,B, ACN treatment significantly reduced angiogenesis. Thus, considering 100% angiogenesis in untreated controls, ACN inhibited angiogenesis in the edges and interior of lung sections by 89.9 ± 11.8% and 81.8 ± 6.6%, respectively (difference between means ± SEM, respectively), as illustrated in Figure 7C.

In addition, Cenpf overexpression has been correlated with poor survival of BC patients. Results showed that Cenpf mRNA levels in lungs were downregulated by ACN to 0.6-fold of untreated control (Figure 7D). However, modulation of Cenpf mRNA did not reach significance (*p* = 0.0768) because of the small sample size and variability between samples. The lowered levels of Cenpf mRNA suggested that ACN may act by reducing the spread of 4T1 cells to lungs.

A summary of the mechanisms modulated by ACN in 4T1 BC cells in vitro and in vivo is presented in Table 1.

## 3. Discussion

There is currently a great need to use dietary approaches to help TNBC patients, who have a very high likelihood of developing recurrence and metastasis within 5 years of diagnosis. Anthocyanins are the most abundant flavonoid constituents of fruits and vegetables and have shown beneficial effects in the treatment of BC [18,19,20,24]. Sweet cherries, especially varieties rich in anthocyanins (DSC), can become a viable dietary chemopreventive and therapeutic option that could be adopted by women. Anthocyanins extracted from DSC have proved to be safe and to effectively reduce tumor growth and multiple pro-oncogenic signaling pathways in hormone-negative and HER2-positive MDA-MB-453 BC cells in vitro [18,19] and in vivo [20].

The main phenolic compounds in DSC and present in WE have been identified [18] as a mixture of phenolic acids (19 were identified), anthocyanins (5 were identified), flavonols (6 were identified), and proanthocyanidins (5 were identified). The DSC extract enriched in anthocyanins (ACN) contained Cyanidin-3-glucoside, Cyanidin-3-rutinoside, Peonidin-3-rutinoside, Malvidin-3-glucoside acetaldehyde, and Cyanidin-3-(6-O—p-coumaroyl)-5-O-diglucoside [18].

In this study, ACN reduced the viability of TNBC 4T1 cells with higher potency than WE (Figure 1A), in agreement with studies reporting the activity of ACN against the HER2+ MDA-MB-453 and TNBC MDA-MB-231 cells [18,19]. The BC cytotoxic effect exerted by ACN can be attributed to the most abundant C3G, cyanidin-3-O-rutinoside, and peonidin-3-O-rutinoside [18].

However, the ACN dose that inhibited cell viability by 50% (IC_50_) and ROS by ~50% (58.6 µg C3G/mL) would be complicated to translate into product dietary intake because it represents the concentrations in target cells, which are used to investigate the cellular mechanisms modulated by such compounds.

Suppression of ROS (as shown in Figure 1B) is beneficial because cancer cells use ROS signals to drive neoplastic manifestations [25]. ROS can promote tumor progression through multiple mechanisms, including the generation of mutations, the alteration of pro-oncogenic signaling cascades, and promotion of stemness in cancer cells [26]. Results from this study are supported by a previous study demonstrating the ROS scavenging capacity of C3G in BC cells overexpressing ErbB2 and its role in blocking the ErbB2/cSrc/FAK pathway needed for cell migration and invasion [27].

Apoptosis is a programmed cell death where various morphological changes occur with complexity and energy dependence [28]. Caspase-3 is the most important executioner caspase, responsible for most of the proteolysis during apoptosis. Caspase-3 plays a key role in PARP inactivation through dissociation of its DNA-binding domain, which, upon binding to a damaged site, prevents other non-cleaved PARP from accessing the damaged site and initiating repairs [27]. In this study, ACN induced caspase-3 cleavage (Figure 2A) accompanied by a decrease in PARP protein levels (Figure 2B). The absence of caspase-8 cleavage strongly suggests the mitochondrial apoptotic pathway as a central mechanism responsible for ACN-induced 4T1 cell death [23]. In this context, the stress-activated p38 phosphorylation could have been a compensatory pathway in response to ACN-mediated ERK1/2 inhibition that contributed to apoptosis [4]. Furthermore, natural antioxidants have been shown to induce apoptosis in 4T-1 cells, mediated at least in part by p38 and caspase-3 activation [29]. ACN also upregulated p-p38 in MDA-MB-453 BC cells, and its inhibition with SB203580 confirmed its contribution to the intrinsic mitochondrial pathway [19]. Then, the ACN can be added to the list of natural dietary compounds that induce the activation of p38-mediated cancer cell apoptosis similarly to a number of chemotherapeutic agents, but without their side-effects [30]. It also appears that the apoptosis induced by ACN-mediated increased cellular stress was accompanied by p-CREB suppression, thus decreasing the transcription of anti-apoptotic genes such as the Bcl-2 [23]. 

In contrast to the p38 pathway, the ERK pathway forms an extensive regulatory network that plays a key role in cancer cell growth and proliferation in 4T1 BC cells [31]. ERK1/2 activation by phosphorylation is closely related to tumor progression and resistance to chemotherapeutic drugs [32]. Results from this study showed the downregulation of ERK2 mRNA levels and cell signaling pathway activation through ERK1/2 phosphorylation (Figure 3A,B). These results are supported by previous studies. For example, polyphenols extracted from muscadine grapes abrogated the ERK1/2 pathway [33]. Anthocyanins extracted from pomegranate also showed reduction in tumor volume in mice through inhibition of ERK1/2 phosphorylation [34]. Delphinidin inhibited cell proliferation through ERK and PI3K/Akt pathways and CREB at the transcriptional level in B16-F10 endothelial cells [35]. The C3G isolated from blackberries downregulated the ERK1/2 pathway in JB6 cells [36]. 

In addition, CREB transcription factor activation, occurring downstream of ERK1/2 and Akt cell signaling pathways, was downregulated by ACN in 4T1 BC cells (Figure 3C). CREB has been implicated in aberrant signal transduction caused by the deregulated expression of downstream genes that control the hallmarks of cancer, such as proliferation, apoptosis, angiogenesis, and metastasis [37].

The mutations in the RAS genes are characteristic of most malignant tumors, with RAS acting as an activator for both the MAPK ERK and PI3K/Akt pathways. Moreover, the dynamic interaction between RAS/ERK and RAS/PI3K/Akt is characterized by both positive and negative feedback loops, involving ERK1/2-PI3K/Akt crosstalk and bidirectional communication with other pathways to mediate multiple cellular functions critical to tumor initiation, progression and outcome [4,38,39].

The ACN-mediated suppression of the Akt pathway (Figure 4), is consistent with a previous study demonstrating that anthocyanins isolated from *Vitis coignetiae* Pulliat (AIMs) also downregulated Akt activation in MCF-7 cells, leading to reduced resistance to cisplatin treatment and increased apoptosis [6]. Akt signaling was also downregulated in TNBC MDA-MB-231 cells by C3G treatment (150–500 μM) along with apoptosis induction [40]. These results have important implications considering that p-Akt phosphorylates a wide range of downstream effectors that regulate apoptosis, transcription factors, and oncogenic factors [41].

Levels of full-length proteins within the Akt/mTOR signaling pathway in 4T1 cells treated with ACN remained without significant change (Supplementary Table S1), except for RPS6 protein. Results shown in Figure 4C support the role of ACN in Akt inhibition since downregulation of total RPS6 has been also reported in TNBC cells upon Akt inhibition with MK2206 [42]. RPS6 overexpression contributes to metastasis of cancers since it enhances cancer cell migration together with upregulation of epithelial–mesenchymal transition (EMT) markers [43]. 

VCAM-1 also promotes cell migration, in part via the Src signaling pathway leading to invasion, migration, and adhesion [44,45]. In this context, results shown in Figure 5A,B suggested that VCAM may be part of the focal adhesion Src signaling pathway leading to cancer cell motility to the wounded area (Figure 5C). These results are supported by studies demonstrating that ACN from black rice suppressed EMT of BC cells (MCF-7, MCF-10A, MDA-MB453 and the TNBC MDA-MB231) by reducing Src phosphorylation [24,27].

Overall, the clinical benefits of Akt-ERK dual inhibition by ACN (Figure 3B and Figure 4A,B) as well as downregulation of the Src signaling pathway confirm our hypothesis that ACN has BC anti-invasive activity and may be promising as a dietary complementary therapy to treat TNBC.

Finally, results from the pilot in vivo study made possible the preliminary assessment of ACN effects on interactions between cancer cells and host. Collected data suggested that ACN may be an alternative for BC patients to treat angiogenesis and prevent metastasis through DSC dietary intake. The ACN dose fed to mice (150 mg/kg/day) showed no adverse effects on animal health in this pilot study as well as in our previous in vivo xenograft study [20]. The 150 mg C3G/kg/day fed to mice would be equivalent to 729.73 mg C3G for a 60 kg human subject [46], which would be supplied by approximately 425 g of DSC edible weight [47]. Conversely, the animal dose (150 mg C3G/kg/day) translated into in vitro dose, would be equivalent to 144 µg/mL (considering the Km value for mouse is 3, 150 mg C3G/kg = 450 mg C3G/m^2^, and the area of a well in a 96-well plate is 0.32 cm^2^; the amount of C3G in each well would be 0.0144 mg, therefore, 0.0144 mg/0.1 mL or 0.144 mg/mL = 144 µg/mL). This concentration is approximately 2.5-fold that of the IC50 (58.6 µg C3G/mL) used for the cell culture experiments. However, ingested compounds are subjected to pharmacodynamics and pharmacokinetics of absorption, distribution, and elimination parameters that will play a role in decreasing the active concentrations in target tissues.

Preliminary results showing downregulation of p-ERK1/2 and p-CREB (Supplementary Figure S4) were consistent with results found in vitro, suggesting a decrease in invasive genes. The overexpression of ERK1/2 has been shown to culminate in CREB phosphorylation and increased transcription of pro-angiogenesis gene vascular endothelial growth factor [VEGF] and cyclooxygenase-2 in BC [48]. Likewise, lowering ERK1/2 phosphorylation in 4T1 tumor-bearing mice had a significant effect on primary tumor outgrowth and lung metastasis [31] and attenuated CREB phosphorylation and tumor cell survival [49].

Feeding the mice with ACN (150 mg/kg/day) for only 1 week reduced angiogenesis in tumors (Figure 6A,B) and lungs (Figure 7A–C). These findings are especially relevant considering that angiogenesis in TNBC largely contributes to tumor cell proliferation, spread and metastasis [50,51].

The activation of the angiogenic switch in tumors gives them the ability to recruit new capillaries, resuming oxygen and nutrient supply to both the angiogenic and surrounding non-angiogenic cells, leading to tumor growth [43,51]. Results from this study are supported by a previous study showing that C3G attenuated angiogenesis in TNBC MDA-MB-231 cells [52]. Likewise, oral administration of anthocyanin-rich extract from black rice (100 mg/kg/day) to BALB/c nude mice bearing MDA-MB-453 cell xenografts significantly suppressed tumor angiogenesis [53].

Moreover, results showing a reduction in Cenpf mRNA levels in lungs after ACN treatment for 1 week (Figure 7D) are encouraging. The mechanisms by which Cenpf expression regulates BC metastasis has been predicted using GEO databases and validated using a BALB/C mouse 4T1 BC model. It was shown that Cenpf regulates the secretion of parathyroid hormone-related peptide (PTHrP) through its ability to activate the PI3K–AKT–mTORC1 pathway [54]. Immunohistochemical (IHC) analyses have shown high Cenpf expression in BC tissues compared to normal tissues, and Cenpf overexpression in target lesions as well [54]. Furthermore, Cenpf expression correlated with markers of aggressive tumor behavior, poor prognosis, and chromosomal instability [55].

Overall, preliminary data collected from the syngeneic BALB/C mouse pilot study encourage a larger scale animal study to investigate DSC anthocyanins as a dietary alternative to help prevent BC spread and invasion to the lungs. This pilot study also allowed the identification of pitfalls and caveats for a full-scale animal study. The tumor cell implantation with 1 × 10^6^ cells/100 μL Growth Factor Reduced Matrigel led to an excessive tumor growth rate. This was a pitfall because tumors reached ~10% of body weight in the ACN group, and ~15% of body weight in the control group. This made impossible to treat the animals with ACN for more than 1 week. For a larger scale study, 4T1 cells injected into mammary pads should not exceed 5 × 10^5^ cells resuspended in 3X diluted Matrigel in PBS instead of 100% Matrigel [56]. A caveat of this pilot study was the risk involved in performing the 4T1 BC cells implantation into mammary fat pad without surgery. A small skin incision using a sterilized scalpel would help to effectively reach mammary pad to optimize tumor cell implantation.

A future study should consider also using 4T1-luc2 cell implantation to assess BC invasion via in vivo bioluminescence. This approach will allow monitoring of metastasis dissemination and progression in the animal even after surgical tumor removal.

## 4. Materials and Methods

### 4.1. Chemicals, Antibodies, and Reagents

Antibodies against phospho-mTOR, total and phospho-Akt (p-Akt), phospho-cAMP response element binding protein (p-CREB), phospho-ERK1/2 (p-ERK1/2), total and phospho-p38 (p-p38), phospho-Src (p-Src), cleaved caspase-3, caspase-3, and poly (ADP-ribose) polymerase (PARP) were purchased from Cell Signaling Technology (Danvers, MA, USA). Western blotting chemiluminescence luminol reagent was purchased from Santa Cruz Biotechnology Inc. (Santa Cruz, CA, USA). Direct-zol™ RNA MiniPrep Plus mRNA extraction kit was purchased from Zymo Research (Irvine, CA, USA). SsoAdvanced™ Universal SYBR^®^ Green Supermix and iScript^TM^ Reverse Transcription Supermix were purchased from BioRad Laboratories (Hercules, CA, USA). Primers for VCAM-1 (forward ACA-GAA-GAA-GTG-GCC-CTC-CAT and reverse TGG-CAT-CCG-TCA-GGA-AGT-G) and GAPDH (forward GTC-TCC-TCT-GAC-TTC-AAC-AGC-G and reverse ACC-ACC-CTG-TTG-CTG-TAG-CCA-A) were purchased from Integrated DNA Technologies (Coralville, IA, USA). Primers for ERK2 and centromere protein F (Cenpf) were purchased from Sigma-Aldrich (St. Louis, MO, USA). Milliplex Map Akt/mTOR Phosphoprotein Magnetic Bead 11-Plex Kit-Cell Signaling was purchased from Millipore (Billerica, MA, USA).

### 4.2. Extraction of WE and ACN 

The DSC concentrated juice batch (kindly supplied by FruitSmart, Inc. Grandview, WA, USA) that was previously used and analyzed by high performance liquid and mass spectrometry (HPLC-MS/MS) as described in Lage et al. (2019) [18] was also used for this study. The quantitative and qualitative phenolic profiles of this DSC concentrated juice and extracted compounds in WE and ACN fractions have been published [18]. DSC concentrated juice (10 mL) was diluted with acidified water (20 mL, pH = 3.5) and loaded into a preconditioned C18 cartridge (55–105 μm, Waters Corp., Milford, MA, USA). The DSC whole extract (WE) was eluted with pH = 3.5 acidified methanol (MeOH) after sugar removal with 50 mL of nanopure water. To obtain the ACN fraction, DSC diluted juice was loaded into a preconditioned cartridge with MeOH and water pH 2.0. The ACN fraction was eluted with 50 mL of 16% acetonitrile at pH 2.0 after sugar removal with 50 mL water, pH = 2.0. The WE and ACN isolated fractions were evaporated using a vacuum centrifuge (Savant SpeedVac, Thermo Fisher Scientific, Asheville, NC, USA) at 45 °C. Afterwards, the extracts were stored under nitrogen atmosphere at −20 °C for future testing in cell culture experiments. WE and ACN dry extracts were dissolved in cell culture medium for cell culture treatments.

For the in vivo experiments, the ACN fraction eluted with 16% acetonitrile was further diluted 3X with water pH = 3.5, loaded into a third preconditioned C18 cartridge, washed with acidified water (50 mL) to remove any trace of acetonitrile, and eluted with 100% MeOH followed by evaporation in a vacuum centrifuge to remove MeOH, diluted with nanopure water and sterile filtered (25 mm, 0.45 μm PES sterile and endotoxin-free filter, Whatman Puradisc^TM^, Pittsburgh, PA, USA) for mouse treatment by gavage.

In each in vitro or in vivo assay performed, the extracts had their phenolic or anthocyanin content quantified. Total polyphenols in WE were quantified spectrophotometrically as μg gallic acid equivalent (GAE)/mL by Folin–Ciocalteu colorimetric micro-method [57]. Anthocyanin concentration was quantified by the pH-differential spectrophotometric method as mg cyanidin 3-glucoside equivalent (C3G)/mL, as reported [58].

### 4.3. In Vitro Study

#### 4.3.1. Cell Culture

The 4T1 murine BC cells, which mimic stage IV human BC, were purchased from the American Type Culture Collection (ATCC, Manassas, VA, USA). Cells were grown in RPMI-1640 medium containing 2 mM L-glutamine, 10 mM HEPES, 1 mM sodium pyruvate, 4500 mg/L glucose, and 1500 mg/L sodium bicarbonate, supplemented with 10% (*v*/*v*) fetal bovine serum (FBS) and 1% (*v*/*v*) penicillin-streptomycin antibiotic mix (ThermoFisher Scientific, Grand Island, NY, USA), and maintained at 37 °C with a humidified 5% CO_2_ atmosphere.

#### 4.3.2. Cell Viability

To evaluate the effect of ACN on cell viability, the in vitro toxicology resazurin-based assay kit was used (Sigma-Aldrich, St. Louis, MO, USA). 4T1 cells seeded in a 96-well plate were allowed to reach approximately 80% confluence before treatment with WE (160–2500 µg GAE/mL equivalent to 20–320 µg C3G/mL) or ACN (10–80 µg C3G/mL) for 48 h. Relative fluorescence units (RFU) were measured at 560 nm and 590 nm excitation and emission wavelengths, respectively, using the FLUOstar Omega plate reader (BMG Labtech, Cary, NC, USA). Cell viability (% of control) was calculated as: [RFU sample/RFU control] × 100.

#### 4.3.3. Evaluation of ROS Production

The 4T1 cells seeded in a 96-well plate were allowed to reach approximately 100% confluence before treatment with WE or ACN (20–320 µg C3G/mL) for 48 h. Production of ROS was assessed with the Carboxy-H2DFFDA probe (10 µM) (Fisher Scientific, Pittsburgh, PA, USA) following the manufacturer’s protocol. Relative fluorescence units (RFU) were measured at 480 nm excitation and 520 nm emission using a FLUOstar Omega plate reader (BMG Labtech, Cary, NC, USA). RFU were normalized to cell density assessed using the Janus green reagent (Sigma-Aldrich, St. Louis, MO, USA). Briefly, cells were fixed with 100 µL of methanol (100%) for 3 min, air-dried, and stained with 100 µL of Janus green (1 mg/mL) for 3 min. Two washes with PBS were performed, and 100 µL of 50% methanol were added to dissolve Janus green crystals. Cell density was proportional to absorbance at 654 nm.

#### 4.3.4. Wound Healing Assay

Cells were seeded on 24-well plates and incubated until reaching approximately 80% confluence. The cell surfaces were scratched with 200 μL sterile tip followed by PBS rinsing to remove debris. Cells were treated with ACN at the dose shown to inhibit cell viability by 50% (IC_50_) in 10% FBS-supplemented culture medium. Cell migration to the wounded area was assessed after 48 h through microphotographs from random fields (*n* ≥ 3) taken with a Keyence BZ-X710 Microscope (40X). The number of cells that migrated towards the wounded area were quantified using ImageJ software (http://imagej.nih.gov/ij/ accessed on 1 February 2020). Data from at least three independent experiments were analyzed with Graph Prism 5.0 (San Diego, CA, USA).

#### 4.3.5. Gene Expression Analyses

4T1 cells were starved overnight and treated with ACN at the concentration needed to inhibit cell viability by 50% (IC_50_) in 2.5% FBS-supplemented culture medium for 24 h followed by mRNA extraction using Quick-RNA-MicroPrep kit (Zymo Research, Irvine, CA, USA).

The reverse transcription Supermix iScript^TM^ (Bio-Rad, Hercules, CA, USA) was used for cDNA synthesis followed by real-time polymerase chain reaction (RT-PCR) amplification using SsoAdvanced^TM^ Universal SYBR^®^ Green Supermix (Bio-Rad, Hercules, CA, USA). Relative mRNA levels were calculated as reported [59] using GAPDH as housekeeping gene.

#### 4.3.6. Protein Expression Analyses

4T1 cells seeded onto 10 cm culture plates were allowed to reach approximately 80% confluence and starved overnight in FBS-free medium followed by ACN treatment (IC_50_) in 2.5% FBS-supplemented culture medium for 48 h. Cell lysates were obtained using xTractor Buffer (Takara Bio Company, Mountain View, CA, USA) supplemented with protease and phosphatase inhibitor cocktail (Thermo Fisher Scientific, Asheville, NC, USA) following the manufacturer’s protocol. Protein concentrations in cell lysates were quantified using Bradford reagent (BioRad, Hercules, CA, USA) according to manufacturer’s protocol.

For Western blots, the lysates (60 μg protein) were subjected to electrophoresis and wet blotting transfer onto 0.46 μm nitrocellulose (Santa Cruz Biotechnology, Inc., Santa Cruz, CA, USA) as detailed [19]. Overnight incubation with primary antibodies was followed by 2h incubation with secondary antibodies. Protein bands were detected with luminal reagent (Santa Cruz Biotechnology, Inc., Santa Cruz, CA, USA) after 1 min of reaction. The band intensities were quantified using the ImageJ software.

For multiplex magnetic bead-based immunoassay, cell lysates were analyzed using the MILLIPLEX Akt/mTOR Magnetic Bead kit (Millipore, Billerica, MA, USA) to quantify insulin receptor (IR); insulin receptor substrate-1 (IRS-1); insulin-like growth factor-1 (IGF-1); phosphatase and tensin homolog deleted on chromosome 10 (PTEN); Akt, mTOR, ribosomal protein S6 kinase beta-1 (P70S6k), ribosomal protein S6 (RPS6), glycogen synthase kinase 3 α (GSK3α) and β (GSK3β); and TSC2 following the manufacturer’s protocol on a Luminex system. Data were analyzed using xPonent 3.1 software (Austin, TX, USA).

#### 4.3.7. In Vivo Pilot Study

A pilot in vivo study was performed with BALB/C mice purchased from Envigo (Houston, TX, USA) to assess the anti-metastatic potential of ACN. Upon arrival to the Comparative Medicine Program at Texas A&M University, the mice were allowed to adjust to their environment for 1 week. 4T1 cells were orthotopically injected into upper right and left mammary fat pads (1 × 10^6^ cells/100 μL Growth Factor Reduced Matrigel, BD Bioscience, San Jose, CA, USA) under isoflurane anesthesia. Tumors were allowed to grow for 9 days before animals were assigned to ACN (*n* = 2) fed 150 mg C3G/kg body weight/day by gavage or untreated controls (*n* = 2).

Tumor size was monitored every other day, tumor volume was calculated as a^2^ × b/2, where “a” and “b” are the shortest and longest perpendicular diameters of the tumor, respectively. After 1 week treatment, animals were terminated by CO_2_ asphyxiation and cervical dislocation. Harvested tumors and lungs were divided in sections for protein, mRNA, and histological analyses. Tissue sections for protein and mRNA analyses were flash-frozen in liquid nitrogen and stored at −80 °C. Tissue sections for histological analysis were placed in 10% neutral buffered formalin for 24 h and maintained in 70% ethanol at 4 °C.

For mRNA analyses, lung tissues were ground with liquid nitrogen followed by mRNA extraction using Direct-zol-RNA-MiniPrep kit (Zymo Research, Irvine, CA, USA) according to the manufacturer’s protocol. Extracted mRNA was used for cDNA synthesis, and relative mRNA levels were determined as detailed in Section 4.3.5.

For protein analyses, tumor tissues were ground with liquid nitrogen and lysed with xTractor buffer according to the manufacturer’s recommendations. Western blot analyses were performed in tissue lysates as detailed in Section 4.3.6.

### 4.4. Histology

For histological analysis, sections of 5 mm thickness of paraffin-embedded tumor and lung tissues were stained with Hematoxylin and Eosin (H&E). Photomicrographs were taken using the Aperio CS2 digital pathology scanner (Leica Biosystems Inc., Buffalo Grove, IL, USA). Quantitative analysis of angiogenesis area was performed with ImageJ software. Data were collected from *n* ≥ 10 random areas per stained section.

### 4.5. Statistical Analysis

Data from cell viability and ROS are mean from six or more replicates ± standard error of the mean (SEM). Significant difference from untreated controls was determined by one-way analysis of variance (ANOVA) followed by Bonferroni test. Data from band intensities determined with ImageJ software are mean from two independent experiments ± SEM. Data from Millipex MAP total Akt/mTOR magnetic bead assay are mean from three replicates ± SEM. Data from mRNA are mean from four or more replicates ± SEM. Data from wound healing assay are mean from four or more replicates ± SEM. Data from angiogenesis areas quantified with ImageJ software from 10 or more random areas photographed in each H&E stained section are mean (*n* ≥ 50 for tumor tissues or *n* ≥ 12 for lung tissues) ± SEM. Statistical significance was determined with unpaired *t* test with Welch’s correction. Data were analyzed with GraphPad Prism 5.0 (San Diego, CA, USA).

## 5. Conclusions

ACN inhibited 4T1 cell viability in a dose-dependent manner with IC_50_ = 58.6 µg C3G/mL and decreased production of ROS. ACN-induced apoptosis was confirmed by caspase-3 cleavage and total PARP decrease. In addition, ACN treatment downregulated ERK1/2 and Akt/mTOR cell signaling pathways and downstream transcription factor activation CREB, which contribute to cell proliferation and invasion. The anti-invasive activity exerted by ACN in 4T1 cells was further demonstrated by suppression of VCAM-1 mRNA and p-Scr protein expression, accompanied by the wound healing inhibition in approximately 88.6%. The in vivo pilot study showed that angiogenesis areas in tumor tissues decreased in 85%. This was accompanied by decreased angiogenesis in lung tissues between 81.8% and 89.9% and lowered Cenpf mRNA levels. DSC may be considered a superfruit due to the great potential exerted by its ACNs as complementary dietary bioactive compounds that may help to fight BC. However, a full pre-clinical animal study is needed to confirm the activity of ACNs in vivo as well as their role in BC treatment and suppression of drug resistance and metastasis.

## Figures and Tables

**Table 1:** Mechanisms modulated by ACN in 4T1 BC cells in vitro and in vivo.

In Vitro Study				
↓ ROS	↑ caspase-3 cleavage	↓ ERK1/2 mRNA	↓ p-Akt protein	↓VCAM mRNA
	↓ Total PARP	↓ p-ERK1/2 protein	↓ p-mTOR protein	
	↑ p-p38/total p38 ratio	↓ p-CREB protein	↓ RPS6 protein	↓ p-Src protein
Decreased oxidative stress	Induced p38-mediated cellular stress and caspase-dependent apoptosis	Downregulated ERK and Akt cell signaling pathways and downstream targets mTOR and CREB involved in transcription of pro-invasive genes, cell growth and migration		Suppressed VCAM-Src cell signaling pathway involved in cancer cell motility
**Pilot in vivo study**				
**Tumors**		**Lungs**		
↓ angiogenesis		↓ Angiogenesis		
↓ p-ERK1/2 protein expression (preliminary)				
↓ p-CREB protein expression (preliminary)		↓ Cenpf mRNA		
Suppressed angiogenesis in tumor tissues, possibly linked to ERK/CREB downregulation		Suppressed angiogenesis in lung tissues and downregulated Cenpf mRNA associated with lung metastasis.		

↓Decrease, ↑Increase.

## Data Availability

The data presented in this study are available on request from the corresponding author.

## Supplementary Materials

The following supporting information can be downloaded at: https://www.mdpi.com/article/10.3390/molecules27217245/s1, Figure S1: ACN did not activate apoptosis cascade within 24 h. Cleaved and total caspase-3 protein levels (A). Total PARP protein levels (B). Cells starved overnight were treated with ACN (IC50) in 2.5% FBS supplemented culture medium for 24 h. Cell lysates were subjected to immunoblot analysis with primary and secondary antibodies as detailed in materials and methods. Western blots are representative of at least 2 independent experiments. Figure S2: PI3K/Akt/mTOR pathway in TNBC. Figure created in BioRender.com. Figure S3: Levels of p-mTOR/Total mTOR ratio in 4T1 BC cells. Cells starved overnight were treated with ACN (IC50) in 2.5% FBS supplemented culture medium for 48 h. Cell lysates were subjected to immunoblot analysis for p-mTOR or multiplex Magnetic Bead immunoassays for total mTOR as detailed in materials and methods. Data from immunoblot band intensities were divided by MFI data to determine p-mTOR/total mTOR ratio. Data (mean (*n* = 2) ± SEM) was analyzed using the t test with Welch’s correction (* *p* < 0.05). Figure S4. Levels of p-ERK1/2 and p-CREB protein in tumor tissues. Protein levels of p-ERK1/2 (A), and p-CREB (B) in tumor tissues excised after 1-week ACN treatment (150 mg C3G/kg BW/day). Tumor tissues grinded with liquid nitrogen and lysed with xTractor buffer were analyzed as detailed in materials and methods. Western blots are representative of at least 2 independent experiments (upper panel). Band intensities of target proteins determined with ImageJ software were divided by band intensity of β-actin and values were normalized to untreated control (lower panel). Data from relative band intensity determined with ImageJ software are mean (*n* = 2) ± SEM. Table S1: Expression levels of total proteins involved in the Akt/mTOR signaling pathway.

## Sample Availability

Samples of the DSC anthocyanins are available from the authors.
