# Supplementary material for: Dark Sweet Cherry (Prunus avium) Anthocyanins Suppressed ERK1/2-Akt/mTOR Cell Signaling and Oxidative Stress: Implications for TNBC Growth and Invasion
Source: Molecules. 2022 Oct 25;27(21):7245. doi: 10.3390/molecules27217245 (PMC9657292; doi:10.3390/molecules27217245)
Supplement: Supplementary file 1 [file molecules-27-07245-s001.zip › molecules-1915062-supplementary.pdf]

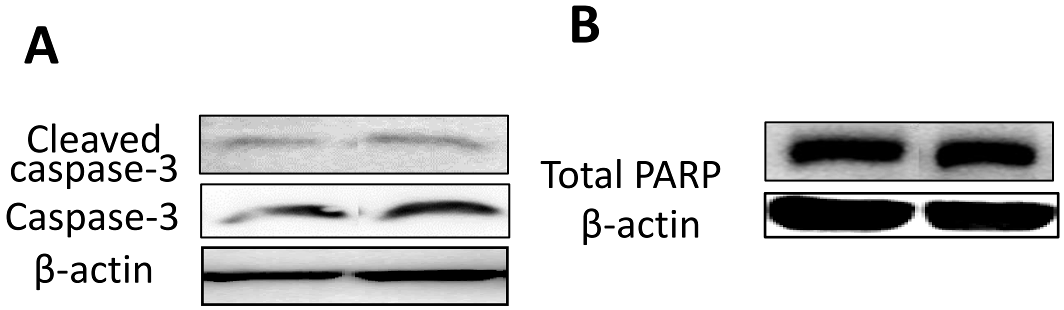

Figure S1. **ACN did not activate apoptosis cascade within 24 h**. Cleaved and total caspase-3 protein levels (A). Total PARP protein levels (B). Cells starved overnight were treated with ACN (IC_{50}) in 2.5% FBS supplemented culture medium for 24h. Cell lysates were subjected to immunoblot analysis with primary and secondary antibodies as detailed in materials and methods. Western blots are representative of at least 2 independent experiments.

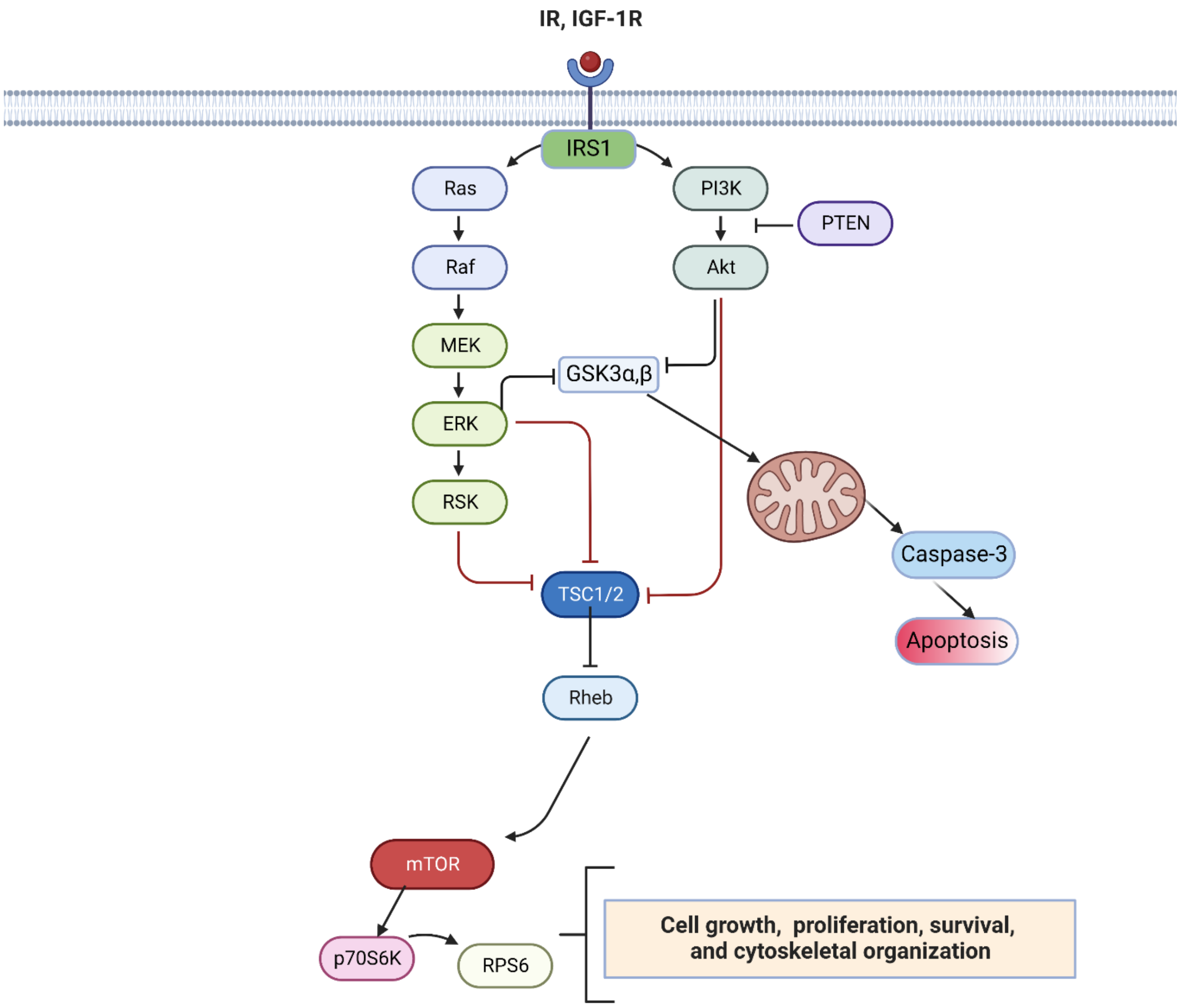

Figure S2. PI3K/Akt/mTOR pathway in TNBC. ***Figure created in BioRender.com.***

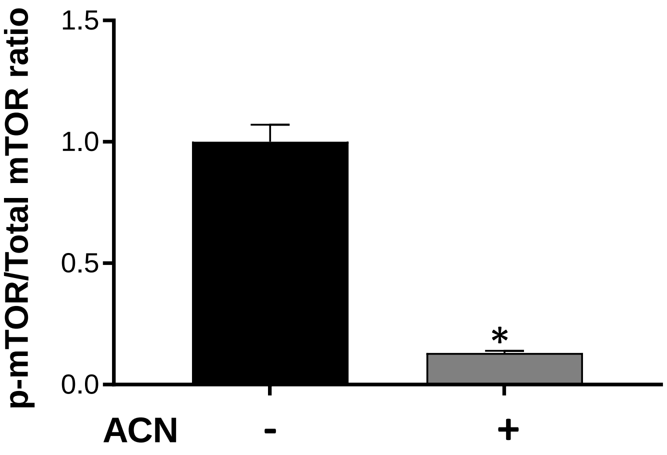

Figure S3. Levels of p-mTOR/Total mTOR ratio in 4T1 BC cells. Cells starved overnight were treated with ACN (IC_{50}) in 2.5% FBS supplemented culture medium for 48h. Cell lysates were subjected to immunoblot analysis for p-mTOR or multiplex Magnetic Bead immunoassays for total mTOR as detailed in materials and methods. Data from immunoblot band intensities were divided by MFI data to determine p-mTOR/total mTOR ratio. Data (mean (n = 2) ± SEM) was analyzed using the t test with Welch's correction (*$p < 0.05$).

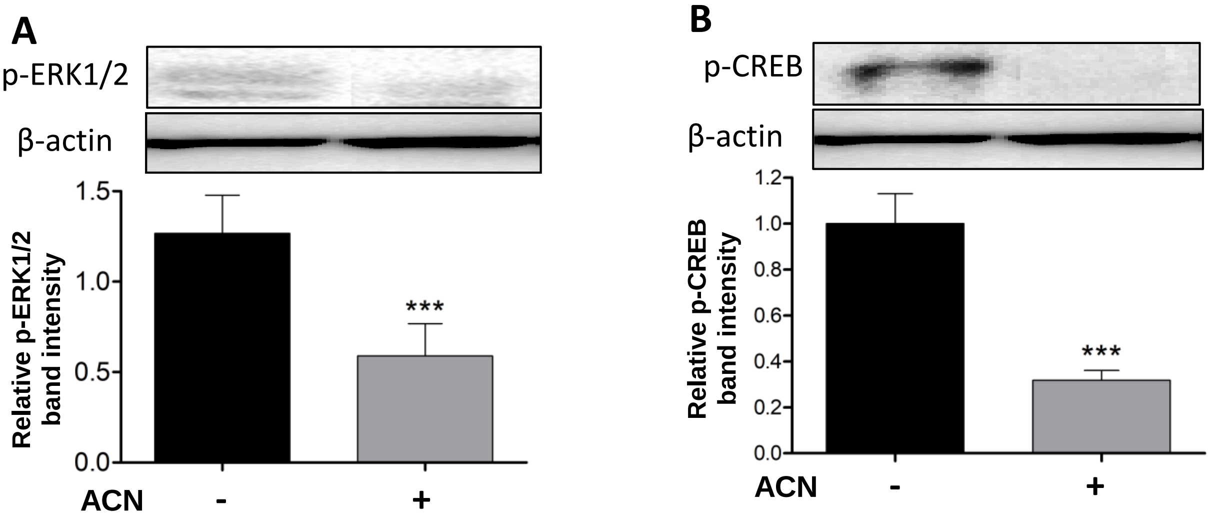

Figure S4. Levels of p-ERK1/2 and p-CREB protein in tumor tissues. Protein levels of p-ERK1/2 (A), and p-CREB (B) in tumor tissues excised after 1-week ACN treatment (150 mg C3G/kg BW/day). Tumor tissues grinded with liquid nitrogen and lysed with xTractor buffer were analyzed as detailed in materials and methods. Western blots are representative of at least 2 independent experiments (upper panel). Band intensities of target proteins determined with ImageJ software were divided by band intensity of β-actin and values were normalized to untreated control (lower panel). Data from relative band intensity determined with ImageJ software are mean (n = 2) ± SEM.

Table S1. Expression levels of total proteins involved in the Akt/mTOR signaling pathway.

Proteins	Control 24 h	ACN 24 h	Control 48 h	ACN 48 h	*P* value
GSK3β	6412 ± 714	5084 ± 217	4805 ± 299	4756 ± 179	NS
IGF-1R	61.5 ± 9.5	60 ± 5.7	50.17 ± 8.4	44.7 ± 7.6	NS
IRS-1	6100 ± 53.8	6384 ± 254	5157 ± 136	5234 ± 98.7	NS
Akt	11072 ± 89.1	10971 ± 99.3	10414 ± 258	10918 ± 226	NS
mTOR	649 ± 49.8	514 ± 20.1	864 ± 19.8	882 ± 60	NS
p70S6K	7665 ± 114	7930 ± 62.6	7252 ± 228	7079 ± 566	NS
IR	11131 ± 182	579 ± 23	734 ± 310	240 ± 52.2	NS
PTEN	9765 ± 192	9773± 79	9137 ± 318	9249 ± 235	NS
GSK3α	30.3 ± 12.4	7.7 ± 1.7	ND	ND	NS
TSC2	2032 ± 50	1812 ± 60	2545 ± 61	2457 ± 27	NS
RPS6	593 ± 18.8	380 ± 18.2	786.2 ± 90[a]	447 ± 20[b]	0.049

Protein levels were analyzed using the MILLIPLEX MAP Total Akt/mTOR Magnetic Bead 11-Plex Kit (Millipore, Billerica, MA, USA). Data are mean (n = 3) ± SEM. The t test with Welch's correction was used to compare ACN 24h and 48h to their respective controls. NS: not statistically significant ($p > 0.05$), ND: not detected. Different superscripts letters within a row indicate statistical significance ($p < 0.05$).
